# Performance feedback and obsessive passion: The moderating role of human capital

**DOI:** 10.1371/journal.pone.0302180

**Published:** 2024-04-18

**Authors:** Sanggeun Lee, Younggeun Lee, Kyung Min Park

**Affiliations:** 1 Whitman School of Management, Syracuse University, Syracuse, NY, United States of America; 2 College of Business and Economics, California State University, Los Angeles, CA, United States of America; 3 School of Business, Yonsei University, Seoul, Korea; KAIST: Korea Advanced Institute of Science and Technology, REPUBLIC OF KOREA

## Abstract

Based on the behavioral theory of the firm, we research how performance feedback influences the formation of CEOs’ passion. We articulate that previous performance directly increases CEOs’ passion. Specifically, positive affective outcomes (performance above aspiration-level) advance CEOs’ positive feelings and motivation toward the domains of success (obsessive passion). For instance, when a firm accomplishes performance objectives, such as reaching sales goals, CEOs’ positive feelings toward developing current products would be boosted. Moreover, negative affective outcomes (performance below aspiration-level) would also positively impact CEOs’ passion since the CEOs would endeavor to reduce the difference between performance and their aspiration-level. As such, performance feedback is a critical antecedent of CEOs’ obsessive passion. In addition, we apply human capital as a moderator between performance feedback and CEOs’ obsessive passion. Results based on multiphase survey data from 189 CEOs of Korean small- and medium-sized enterprises indicate that both positive and negative performance feedback positively increase CEOs’ obsessive passion. Moreover, human capital negatively moderates the relationship between positive performance feedback and CEOs’ obsessive passion and positively moderates the relationship between negative performance feedback and CEOs’ obsessive passion.

## Introduction

CEOs’ passion has been identified as an important factor in building a successful company [1,2]. CEOs’ passion has been positively related to competencies, competitive strategies [1], innovation [3], and organizational growth [4,5]. Although passion has been linked to important outcomes, there are only a handful of studies that examined antecedents of passion [6,7]. Acknowledging the benefits that CEOs’ passion may bring about to firms, it is critical to understand what may cause CEOs to develop their passion. This paper aims to study how CEOs’ affective state on firm performance influences the formation of passion. Firm performance offers an important roadmap for the CEOs’ future strategy. For instance, strategic leaders perceive negative performance as a potential threat, identify problems, search for solutions, and eventually make organizational changes [8]. Indeed, firms with disappointing performance tend to engage in innovation [9] and strategic changes [10,11]. More importantly, previous studies have found the importance of CEOs’ affective states on firm performance on their behavioral changes. Scholars examined that information related to failure can make individuals insecure, and such affective changes motivate them to improve their situation [12]. Moreover, a firm’s unsatisfactory performance motivates CEOs to seek advice from people with a similar background [13].

Whilst there are various affective factors impacting the behavioral change and decisions of CEOs, we examine how performance feedback impact the changes in CEOs’ passion. Specifically, we focus on CEOs’ obsessive passion as the outcome of performance feedback. Obsessive passion is defined as “a controlled internalization of an activity in one’s identity that creates an internal pressure to engage in the activity that the person likes” [14; p. 756]. In other words, a person with high obsessive passion goes through the process of compulsive internalization of a specific activity to the person’s identity. Moreover, obsessive passion is formed through external factors such as reputation and social acceptance [15]. Hence, performance feedback can act as the external source through which obsessive passion can be formed [12,13].

Specifically, we argue that performance satisfaction over aspiration-level (i.e., positive performance feedback) and under aspiration-level (i.e., negative performance feedback) will increase CEOs’ obsessive passion. Positive performance feedback would enhance CEOs’ affection for their business and motivate CEOs to persist in the current area. Analogously, negative performance feedback would make CEOs feel unstable about their current status and endeavor to concentrate more on improving current domains, which would increase CEOs’ obsessive passion.

Moreover, we adopt human capital as a moderator between performance feedback and obsessive passion. Coff [16] defined human capital as the knowledge, skills, and abilities embodied in individuals. Scholars emphasized the importance of education-level and various experience of CEOs and executives for organizational development [17,18]. Contemplating that human capital influences a firm’s behavior, we state that the impact of performance feedback on obsessive passion would be positively magnified when firms attain high human capital.

In this regard, human capital, encompassing the education and experience of CEOs, serves as a critical lens through which performance feedback is interpreted and acted upon. This knowledge and expertise influence CEOs’ decision-making processes and their emotional engagement with their work, underscoring its role in moderating the relationship between performance feedback and obsessive passion. The integration of human capital acknowledges its capacity to enhance CEOs’ confidence and strategic flexibility, particularly in responding to diverse performance feedback outcomes. Consequently, the influence of human capital on shaping CEOs’ cognitive and affective responses is pivotal, thus positioning it as an essential moderator in understanding the dynamics between performance feedback and obsessive passion.

## Literature review

### Performance feedback

According to the behavioral theory of the firm, CEOs compare the actual performance of the firm against their aspirations when deciding whether to modify existing strategies [8]. Aspiration-level refers to the standard of performance that a firm seeks to achieve. In other words, aspiration-level is organizational target or goal, often influenced by past performance and the performance of comparable firms [8]. The concepts of performance and performance feedback, though interconnected, are distinct in organizational contexts. Performance refers to the actual outcomes achieved by an individual, team, or firm, such as productivity, profitability, and achievement of strategic objectives. Performance feedback, on the other hand, is the information provided regarding the assessment of performance. It involves the evaluation of actual performance against certain criteria or goals. This feedback can be positive or negative and is intended to guide future behavior and decision-making.

Depending on the disparity between actual performance and the previous aspiration-level, the CEOs’ decision varies. Especially when firm performance declines, CEOs initiate to identify the cause [8]. Through this problemistic search, CEOs seek to find solutions for the decline in the performance. When the problem is not severe, CEOs make familiar or small changes [19]. Conversely, if the problem is severe and the gap between the actual outcome and aspiration-level is large, CEOs tend to make radical amendments [19]. Thus, an aspiration-level is considered the standard for CEOs to search for certain solutions or changes for the future strategy [11,20].

Studies have continuously found that performance feedback triggers the change in CEOs’ behavior and decisions. For instance, studies found that firms with positive performance feedback decrease radical changes. Such firms decrease their R&D intensity [21], new product introduction [22], innovation development [9], and organizational changes [23]. Firms with positive performance feedback refrain from making changes in their behavior and try to maintain their performance. Since firms’ previous strategies or behaviors have been a success, CEOs tend not to make radical changes or alter their strategies. In the situation of negative performance feedback, different patterns of changes in CEOs’ behaviors have been observed. For instance, scholars found that negative performance feedback motivates CEOs to make organizational changes [23], increase their risk-taking actions [10,13,23,24], display advice search behavior [13], take corporate illegal actions [25], and conduct R&D search behaviors [26]. These studies indicate that negative performance stimulates CEOs to find better strategies to overcome the undesired situations.

Scholars have integrated multi-level approaches into the performance feedback literature by recognizing the interplay of complex factors that shape CEOs’ reactions and decision-making processes. This nuanced understanding acknowledges that responses to performance feedback are influenced not only at the organizational-level but also by individual-level dynamics, where personal characteristics and organizational context significantly affect outcomes. Research in this domain has shown how personal experiences, cognitive biases, and organizational power structures interact with performance feedback. For instance, scholars examined how individual aspirations and organizational identification influence strategic behaviors, underscoring the importance of personal and social dynamics in response to feedback [27–29]. Similarly, scholars further studied how CEOs’ perceptions, such as compensation and power within their organizations, shape their strategic decisions in response to inconsistent feedback, highlighting the complex interplay between individual agency and organizational structure [30,31]. We advance this line of literature by examining the interplay between performance feedback and firm-level human capital in shaping individual-level passion.

### Obsessive passion

Passion is a strong preference toward a specific activity that individuals consider important and pursue with persistence [14]. Vallerand et al. [14] established the dualistic model of passion, which delineates two distinct forms of passion: harmonious and obsessive. Harmonious passion arises from an autonomous internalization of an activity into one’s identity, characterized by a voluntary inclination towards the activity [14]. Obsessive passion results from a controlled internalization of an activity, where engagement is driven by external pressures [14]. Both types of passion involve the integration of the activity into the individual’s identity, highlighting the significant role the activity plays in how they define themselves [15]. Regardless of the type of passion, individuals demonstrate a long-term, enduring commitment to the activity, investing time and energy into its pursuit [14].

Harmonious passion is rooted in autonomous motivation, where the individual engages in an activity out of genuine interest and enjoyment [32]. Conversely, obsessive passion stems from controlled motivation, driven by external pressures and contingent self-esteem, leading to engagement felt as compulsory [15]. Furthermore, individuals with harmonious passion engage in activities with flexibility and volition, capable of prioritizing and integrating their passion within the broader spectrum of their lives [32]. Meanwhile, those with obsessive passion exhibit a rigid persistence in their activities, often with a lack of control over engagement [15].

Specifically, people with obsessive passion experience intense emotions directed toward a particular domain. Scholars have examined various antecedents of obsessive passion. For instance, affective interpersonal commitment [33], job fit [34], and role overload [35] positively increase obsessive passion. Further, obsessive passion is influenced from external motivations such as reputation, social acceptance, and monetary rewards [15].

In the research of CEOs, scholars have delved into how CEOs’ passion impacts organizational outcomes. Foundational papers on passion have found that CEOs’ passion for work significantly enhances organizational performance and growth, primarily through their industry expertise and motivational factors like vision and goals [1,4]. However, contrasting findings point out no empirical correlation between CEOs’ obsessive passion and growth in sales or profits [36]. Furthermore, studies demonstrate a positive association between CEOs’ passion and innovation, particularly in terms of radical and technological advancements and firm-level entrepreneurial orientation [3,37,38].

This paper expands the antecedent studies of obsessive passion by studying performance feedback. From various types of passion, we specifically chose obsessive passion due to its theoretical applicability in examining external pressures such as firm performance [6,15,39]. As obsessive passion is an emotion formed by irresistible and uncontrollable internalization often caused by external sources of motivation [14], we state that performance aspiration and satisfaction would be highly associated with obsessive passion. Supporting this notion, Cardon and Kirk [40] explain how performance feedback can provoke entrepreneurs to persist in their passion domains, and call for studies on the relation between performance feedback and passion. Consequently, this research aims to explore how CEOs’ obsessive passion fluctuates based on different types of performance feedback, thereby contributing to the broader understanding of how external factors like performance feedback impact CEO behaviors and emotional state.

## Hypotheses development

### Performance feedback and obsessive passion

Based on the performance feedback and passion literature, we claim that previous performance would influence CEOs’ obsessive passion because positive outcomes would lead CEOs to feel stronger affection toward current domain, and negative outcomes would pressure CEOs to concentrate more on the domain to change the status quo. When a firm’s performance is above aspiration-level, CEOs tend to make conservative decisions to maintain their success and growth [41–43]. In other words, when CEOs feel satisfaction from firm performance, they tend to reinvest in their current domains and focus on developing existing strategies. As CEOs achieve their organizational goals, they tend to make decisions to protect what firms have gained than to expand new domains [44]. This is because performance achievement is a signal to the CEOs that they have succeeded with their strategies, which leads them to feel strong affection toward their domains of work [34,45]. Although the success may not be entirely emanated from the strategies employed by the CEOs, the self-enhancement process can lead to a biased interpretation of performance feedback [46]. In other words, CEOs might interpret positive feedback as a validation of their strategies and place undue weight on positive feedback, leading to overconfidence and similar decisions on the current domains (i.e., obsessive passion).

Based on the performance feedback literature, scholars explain the impact of self-enhancement in how CEOs set performance aspirations [47]. Specifically, CEOs may set overly ambitious goals based on an inflated sense of past performance (i.e., positive performance feedback), potentially setting the stage for repetitive and fixated decisions. In other words, performance above aspiration-level will motivate CEOs to concentrate on their current successful domains. Moreover, self-enhancement can hinder organizational learning and adaptation by creating a false sense of security [46]. When faced with positive performance feedback, CEOs might fail to recognize the need for change, leading to stagnation and eventual compulsive feelings toward their domains (i.e., obsessive passion). Therefore, we hypothesize that:

Hypothesis 1. *Positive performance feedback (i*.*e*., *performance satisfaction above aspiration-level) is positively associated with CEOs’ obsessive passion*.

CEOs’ obsessive passion would also be increased when performance is below aspiration-level. As problemistic search occurs to handle negative performance feedback [8], CEOs will find a way to reduce the gap between actual performance and their aspiration-level. For instance, CEOs might concentrate on strategy modification [19], increase R&D investments [48], or make product innovation [9]. In this regard, CEOs would increase their passion to modify previous dissatisfied results. When CEOs confront failure, they tend to experience negative emotions [49] and feel insecure about their tenure, thus make an effort and work hard to change the status quo [12]. As CEOs’ endeavor continues, passion would be nurtured [50]. When CEOs confront negative performance feedback, CEOs would invest more time and persist on work to avoid further failures, increasing obsessive passion. Similarly, Cardon and Kirk [40; p. 1042] state that “entrepreneurial passion may be more relevant to persistence especially in the face of negative performance feedback”. Moreover, CEOs take reparative actions when negative emotions, such as guilt, occur from negative performance [51,52]. In other words, these CEOs would concentrate more and deliberately work on their domain to reduce the performance discrepancy.

Building on the performance feedback literature, scholars examined how CEOs exhibiting self-enhancement tendencies resist necessary changes even in the face of negative feedback [53]. This resistance can stem from a belief in the inherent rightness of their current strategies and an underestimation of the challenges they face. In other words, when influenced by self-enhancement, CEOs might ignore or downplay negative feedback, leading to missed opportunities for learning and improvement (e.g., biased toward their original domains without further changes) [46,54]. Therefore, we hypothesize that:

Hypothesis 2. *Negative performance feedback (i*.*e*., *performance satisfaction below aspiration-level) is positively associated with CEOs’ obsessive passion*.

### The moderation effect of human capital

Human capital theory focuses on the economic value of education and experience. Human capital is defined as skills or knowledge that are advantageous for certain decisions [17,55]. Scholars highlighted the value of human capital based on the knowledge-base it offers for decision-making process [56,57]. Scholars applied human capital in the management research. For instance, previous findings show that high level of human capital leads to competitive advantage [58,59], organizational performance [60], success [e.g., 61,62], and venture survival [63]. Moreover, human capital would lead to certain organizational behaviors such as firm flexibility [59], internationalization [64], and entrepreneurial orientation [65]. Overall, scholars revealed that high level of human capital would bring positive outcomes to the firm.

Based on the literature, a high level of human capital is acknowledged to influence both firm- and individual-level factors [62]. In this paper, we articulate that human capital would moderate the relationship between performance feedback and obsessive passion. Specifically, a high level of human capital would strengthen both the relationships between positive performance feedback-obsessive passion and negative performance feedback-obsessive passion. This is because human capital acts as an instruction for managerial decisions and increases CEOs’ confidence with accumulated know-how on organizational outcomes.

First, human capital represents a knowledge corridor where CEOs depend on their previous education and experience to make major decisions [66]. As CEOs analyze their previous positive performance outcomes, they are likely to develop higher passion toward the domains of their focus especially when firms have experienced and well-educated executives. Knowledge empowers CEOs to understand and evaluate their organization’s performance outcomes [67]. This understanding, in turn, invigorates their emotional responses. Moreover, when CEOs gain high satisfaction from their performance outcomes, human capital would positively intensify CEOs’ passion because of knowledge dependency. In other words, human capital would increase the rigid decision-makings when firms are doing well and eventually lead CEOs to concentrate more on their passion of domain.

This also applies to the relationship between negative performance feedback and CEOs’ obsessive passion. Firms with high human capital have instructions on various organizational circumstances including negative performance. Based on their know-how gained from human capital, CEOs understand to be more flexible and gain feedback from others to improve firm performance [68]. In other words, when CEOs are disappointed with their performance and face high gap between their performance aspiration and satisfaction, firms with high human capital are more likely to invest more resources on the current domain, eventually increasing CEOs’ obsessive passion. As such, we argue that human capital would strengthen the positive impact of dissatisfied performance results on CEOs’ passion.

Second, human capital promotes CEOs’ confidence with collective know-how on various organizational management [69,70]. When CEOs achieve positive results, their passion becomes heightened, especially when they have a high level of confidence in their decisions [40]. Firms with high human capital show tendency to imitate previous successful decisions [71]. In other words, CEOs tend to reiterate their decisions when they experience a high level of satisfaction with firm performance and prioritize reinforcing what aligns with their emotional preferences.

Moreover, firms with high human capital, drawing upon their accumulated expertise and know-how, tend to make cautious decisions when confronted with adverse outcomes. CEOs with diverse experiences possess knowledge related to performance management. Empowered by this knowledge, when these CEOs encounter challenging situations related to firm performance, they are more inclined to search for solutions and opportunities for performance improvement, resulting in a heightened passion for the specific area. In other words, the positive influence of performance dissatisfaction on CEOs’ obsessive passion would be intensified in firms with high human capital. As such, we hypothesize that a firm’s high level of human capital would magnify the performance feedback and obsessive passion relationships.

Hypothesis 3. *The greater human capital*, *the stronger positive effect of positive performance feedback (i*.*e*., *performance satisfaction above aspiration-level) on CEOs’ obsessive passion*.
Hypothesis 4. *The greater human capital*, *the stronger positive effect of negative performance feedback (i*.*e*., *performance satisfaction below aspiration-level) on CEOs’ obsessive passion*.

## Methods

### Sample

To test the hypotheses, we utilized multiphase (i.e., six-month lagged) survey data collected from 189 CEOs of Korean small- and medium-sized enterprises (SMEs). In SMEs, the organizational structure typically features fewer hierarchical levels. This structure positions the CEOs in a pivotal role in the daily management of these firms [72]. Such a setting enables CEOs of SMEs to interact closely with both mid-level managers and frontline employees. As a result, these CEOs often have increased discretion in organizational decision-making, compared to CEOs of larger firms. The streamlined hierarchies in SMEs afford their CEOs a wider array of strategic choices and more accessible resources. Moreover, these CEOs frequently handle a diverse range of organizational matters [73]. Therefore, examining the impact of organizational-level performance on individual CEOs is particularly relevant in the context of SMEs.

The list of firms was obtained from the Ministry of SMEs and Startups of Korea. In the first phase (i.e., July 2019), we sent an online survey to 1,268 CEOs to inquire questions on independent (i.e., performance aspiration) and control variables (i.e., firm- and individual-level factors); as a result, we received 486 responses. In the second phase (i.e., January 2020), we sent an online survey to 486 CEOs to obtain data on independent (i.e., performance satisfaction), moderating (i.e., human capital), and dependent variables (i.e., obsessive passion). This multiphase design was applied to alleviate common method bias and secure robust causal inferences [74–76]. To increase the response rate of the second phase, we offered a $20 donation to an international charity organization per respondent and established a short survey design; as a result, we received 237 responses. We excluded incomplete responses and responses from other than CEOs (e.g., vice-presidents, executives in other positions, and directors). The final sample size was 189 CEOs (i.e., a response rate of 14.9 percent). We used double-back translation to ensure precise translation of survey questionnaires from English to Korean [77] and utilized a seven-point Likert scale for main variables (i.e., performance feedback, human capital, and obsessive passion). The complete version of the survey questionnaires is included in the Appendix. In compliance with ethical standards, this study’s protocols involving human participants were reviewed and approved by the Institutional Review Board at Iowa State University (IRB ID: 19–329). The participants provided their written informed consent to participate in this study.

### Measures

#### Independent variable

To measure performance feedback, we used an 18-item scale from Gupta and Govindarajan [78]. This scale has been widely adopted in strategic management and entrepreneurship research [e.g., 79–81]. The measure includes nine items on performance aspiration and nine items on performance satisfaction. Specifically, questions on performance aspiration ask CEOs’ levels of goals for nine performance criteria; questions on performance satisfaction ask CEOs’ levels of contentment for the same nine performance criteria: total revenue, sales growth, return on assets, return on equity, return on investment, operating profits, cash flow, market share, and ability to fund growth from profits. Both performance aspiration (*α* = .96) and performance satisfaction (*α* = .96) showed a high reliability. In order to operationalize performance feedback, performance aspiration was subtracted from performance satisfaction (i.e., performance satisfaction–performance aspiration). We adopted this subtraction formula to capture the discrepancy between CEOs’ satisfaction on performance and their aspiration-level, which has been continuously utilized in the performance feedback literature [e.g., 30,82]. In this regard, positive performance feedback indicates performance satisfaction above aspiration-level and negative performance feedback implies performance satisfaction below aspiration-level.

#### Moderating variable

To measure human capital, we used a nine-item scale from Jin et al. [59]. Sample items include ‘our managers have knowledge of the strengths and weaknesses of our firm’ and ‘our workers have experience that is relevant to their jobs’. The measure displayed a satisfactory reliability (*α* = .88).

#### Dependent variable

To measure obsessive passion, we utilized a six-item scale from Vallerand et al. [14]. These survey items have been broadly employed and validated in diverse domains and cultural settings [e.g., 15,83]. Following the directions from seminal studies on passion [14,84], we instructed the main domains of activity as CEOs’ entrepreneurial works. For example, sample items include ‘I have almost an obsessive feeling for my entrepreneurial works’ and ‘I have difficulties controlling my urge to do my entrepreneurial works’. The measure displayed an acceptable reliability (*α* = .80).

#### Control variables

We included nine firm- and individual-level factors that potentially influence the model. Regarding the firm-level influences, we controlled for four variables: firm age, firm size, firm type, and slack resources. Firm age was measured as the number of years since the firm’s establishment. Firm size was measured as the number of full-time employees. Firm type was measured as a categorical variable whether the firm is public, private, or others. Slack resources were measured using a four-item scale from De Luca and Atuahene-Gima [85]. For example, an item includes ‘our firm has a large amount of resources available in the short run to fund our initiatives’. The scale indicated an adequate reliability (*α* = .83).

Regarding the individual-level influences, we controlled for five variables: CEO gender, CEO tenure, CEO experience, CEO effort, and CEO entrepreneurial self-efficacy. CEO gender captures whether the CEO is male or female. CEO tenure indicates the number of years the CEO has worked for the current firm. CEO experience denotes whether the CEO has a founder experience or not. CEO effort was captured as the level of effort the CEO has invested in work by a seven-point Likert scale. CEO entrepreneurial self-efficacy was measured using a four-item scale from Zhao et al. [86]. The scale captures CEOs’ confidence level in entrepreneurial activities such as ‘creating new products or services’. The scale demonstrated good reliability (*α* = .88).

We conducted t-tests between respondents and non-respondents in the first phase, and found insignificant differences (e.g., performance aspiration: mean difference = .03, *p* = .73), easing the issue of non-response bias. We tested for the overall model fit applying confirmatory factor analysis, and found that the hypothesized model reveals a satisfactory fit (*χ*^2^[6] = 20.23; CFI = .97; SRMR = .05) compared to a single-factor model (*χ*^2^[11] = 197.06; CFI = .63; SRMR = .22). The chi-square testing also revealed a better fit for the hypothesized model than the single-factor model (Δ*χ*^2^[5] = 176.83, *p* < .001). Moreover, the loading of each item was statistically significant (*p* < .001), and construct reliabilities and average variance extracted presented satisfactory results based on the suggested thresholds [87].

## Results

Table 1 displays the descriptive statistics and correlations of all the variables in the study. The average firm age was about 19 years old, and the average firm size was about 48 employees, representing matured and small-sized firms. Most of the CEOs in the sample were male (93 percent). On average, CEOs had 14 years of tenure at the current firm, and 57 percent of the CEOs had firm founding experience.

**Table 1:** Descriptive statistics and correlations table.

Variables	*N*	Mean	*SD*	1	2	3	4	5	6	7	8	9	10	11	12
1. Firm Age	189	18.64	13.09												
2. Firm Size	189	47.80	118.20	.22**											
3. Firm Type^a^	189	2.52	0.57	-.09	-.25**										
4. Slack Resources	189	3.63	1.18	.01	-.09	-.05									
5. CEO Gender^b^	189	0.93	0.25	-.03	.03	-.04	-.02								
6. CEO Tenure	189	14.29	10.10	.62**	.04	-.10	.09	.04							
7. CEO Experience^c^	189	0.57	0.50	-.25**	-.17*	-.05	.05	.27**	.01						
8. CEO Effort	189	4.44	1.42	-.03	-.05	.02	.23**	-.12	-.09	.06					
9. CEO Entrepreneurial Self-Efficacy	189	5.31	0.99	-.25**	-.10	-.08	.08	.06	-.11	.29**	.19**				
10. Positive Performance Feedback	106	0.53	0.53	.00	.02	.11	-.09	.10	.07	-.02	-.05	-.05			
11. Negative Performance Feedback	83	-0.58	0.55	.10	.06	.11	-.08	-.02	-.02	-.02	-.14	-.18	N/A		
12. Human Capital	189	5.17	0.79	-.14	-.03	-.05	.18*	.03	.00	.12	.14	.27**	.03	-.02	
13. Obsessive Passion	189	3.74	1.16	.20*	.00	.03	-.07	-.03	.18*	-.02	-.16*	.04	.29**	-.15	-.01

Note. * *p* < .05

** *p* < .01.

a. Firm Type coded as (1) Public, (2) Private, (3) Other.

b. CEO Gender coded as (0) Female, (1) Male.

c. CEO Experience coded as (0) No, (1) Yes.

We conducted an ordinary least squares (OLS) regression to test the hypotheses. To test Hypothesis 1, we utilized a sample of 106 CEOs with positive performance feedback. Table 2 presents the regression results for Hypothesis 1. Model 1 contains only control variables, and Model 2 includes controls and the main independent variable (i.e., positive performance feedback). As a result of the OLS regression, positive performance feedback is positively associated with CEOs’ obsessive passion (Model 2, Table 2; *β* = 0.47, *p* < .05), supporting Hypothesis 1.

**Table 2:** Regression results: Positive performance feedback.

	Obsessive Passion			
	Model 1	Model 2	Model 3	Model 4
**Control Variables**				
Firm Age	0.00(0.01)	0.00(0.01)	0.00(0.01)	0.00(0.01)
Firm Size	0.00(0.00)	0.00(0.00)	0.00(0.00)	0.00(0.00)
Firm Type	0.29(0.22)	0.21(0.22)	0.27(0.22)	0.35(0.22)
Slack Resources	-0.17(0.09)	-0.14(0.09)	-0.16(0.09)	-0.17(0.09)
CEO Gender	-0.31(0.41)	-0.48(0.41)	-0.45(0.41)	-0.43(0.40)
CEO Tenure	0.02(0.02)	0.01(0.02)	0.01(0.02)	0.02(0.02)
CEO Experience	-0.28(0.26)	-0.28(0.25)	-0.30(0.25)	-0.35(0.24)
CEO Effort	-0.01(0.08)	-0.02(0.08)	-0.02(0.08)	0.01(0.08)
CEO Entrepreneurial Self-Efficacy	0.20(0.12)	0.22(0.12)	0.18(0.12)	0.19(0.12)
**Independent Variable**				
Positive Performance Feedback		0.47(0.20)*	0.43(0.20)*	3.39(1.38)*
**Moderating Variables**				
Human Capital			0.24(0.15)	0.55(0.21)**
Positive Performance Feedback				-0.56(0.26)*
× Human Capital				
**Constant**	2.68(1.07)*	2.69(1.05)*	1.54(1.27)	-0.51(1.56)
***R* ^2^**	.12	.17	.20	.24
**Δ *R*^2^**		.05	.02	.04

Note. *N* = 106; * *p* < .05

** *p* < .01.

Unstandardized coefficients reported; Standard errors in parentheses.

**Fig 1:**
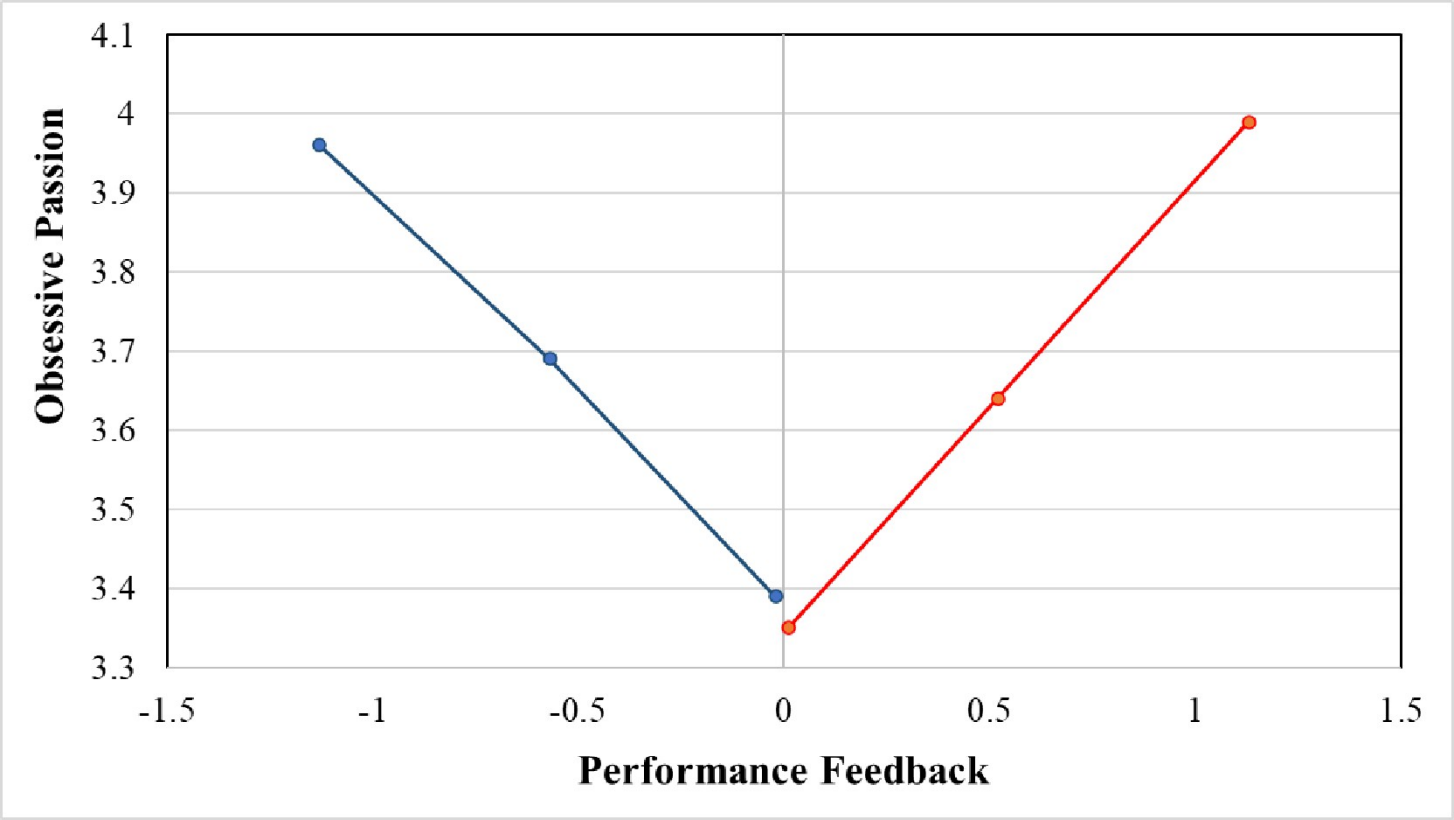
Performance feedback and obsessive passion.

To test Hypothesis 2, we used a sample of 83 CEOs with negative performance feedback. Table 3 presents the regression results for Hypothesis 2. Model 5 contains only control variables, and Model 6 includes controls and the main independent variable (i.e., negative performance feedback). As a result of the OLS regression, negative performance feedback is positively associated with CEOs’ obsessive passion (Model 6, Table 3; *β* = -0.58, *p* < .05), supporting Hypothesis 2. Fig 1 displays the OLS regression results of the relationships between performance feedback and obsessive passion.

**Table 3:** Regression results: Negative performance feedback.

	Obsessive Passion			
	Model 5	Model 6	Model 7	Model 8
**Control Variables**				
Firm Age	0.01(0.02)	0.02(0.02)	0.01(0.02)	0.02(0.02)
Firm Size	0.00(0.00)	0.00(0.00)	0.00(0.00)	0.00(0.00)
Firm Type	0.09(0.28)	0.19(0.28)	0.18(0.28)	0.09(0.27)
Slack Resources	-0.02(0.14)	-0.05(0.14)	-0.02(0.15)	0.01(0.14)
CEO Gender	-0.66(0.85)	-0.70(0.83)	-0.65(0.83)	-0.85(0.82)
CEO Tenure	0.01(0.02)	0.01(0.02)	0.01(0.02)	0.01(0.02)
CEO Experience	0.59(0.36)	0.63(0.35)	0.63(0.35)	0.54(0.34)
CEO Effort	-0.24(0.12)*	-0.29(0.12)*	-0.27(0.12)*	-0.28(0.12)*
CEO Entrepreneurial Self-Efficacy	-0.08(0.17)	-0.13(0.16)	-0.09(0.17)	-0.10(0.17)
**Independent Variable**				
Negative Performance Feedback		-0.58(0.27)*	-0.56(0.27)*	3.27(1.93)
**Moderating Variables**				
Human Capital			-0.15(0.21)	-0.55(0.29)
Negative Performance Feedback				-0.74(0.37)*
× Human Capital				
**Constant**	5.03(1.63)**	4.96(1.58)**	5.31(1.66)**	7.88(2.07)***
***R* ^2^**	.12	.18	.18	.23
**Δ *R*^2^**		.06	.01	.05

Note. *N* = 83; * *p* < .05

** *p* < .01.

Unstandardized coefficients reported; Standard errors in parentheses.

**Fig 2:**
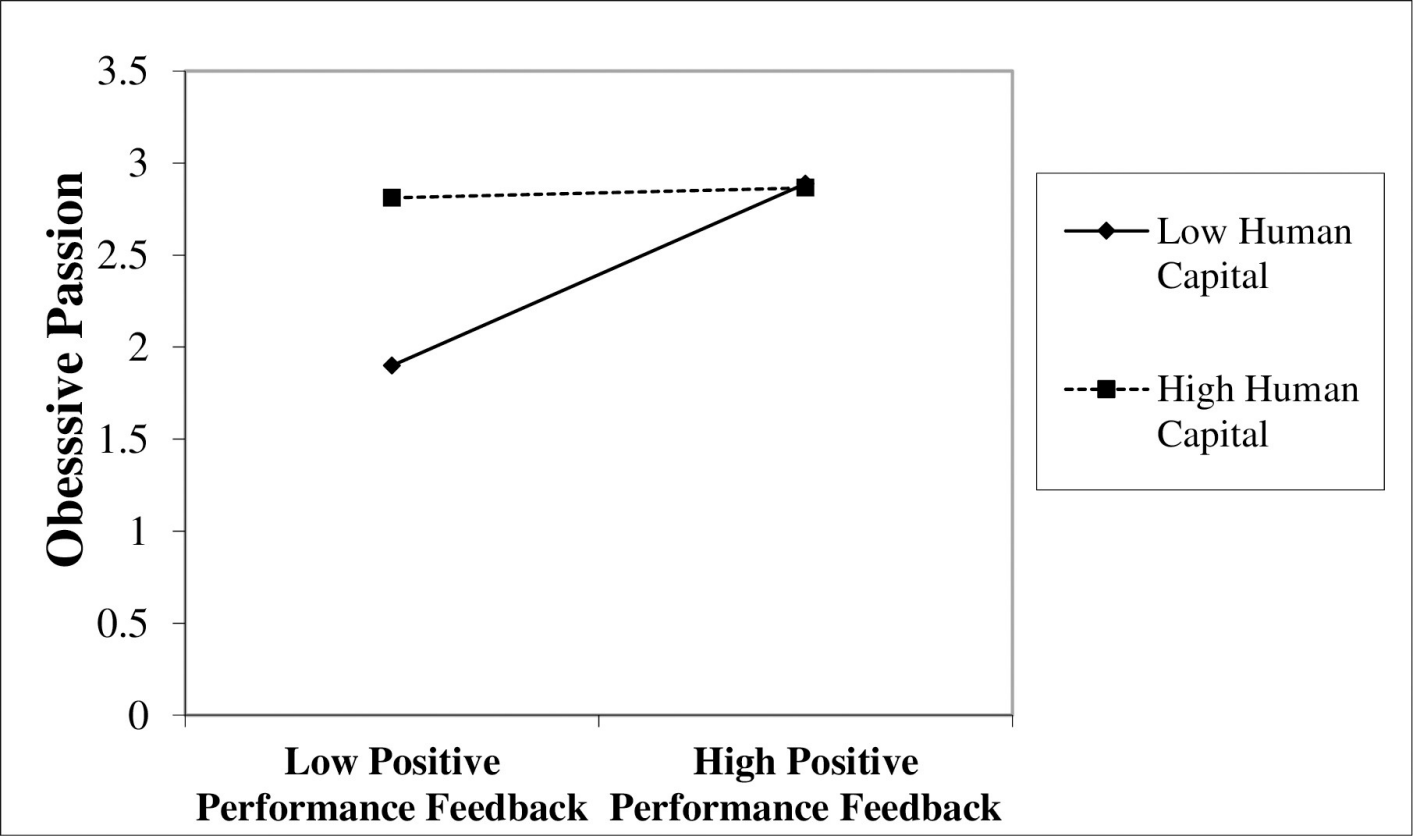
The moderating effect of human capital on the relationship between positive performance feedback and obsessive passion.

To test Hypothesis 3, we adopted a sample of 106 CEOs with positive performance feedback. Table 2 shows the moderated OLS regression results for Hypothesis 3. Model 3 adds the moderating variable (i.e., human capital), and Model 4 contains the interaction variable (i.e., positive performance feedback × human capital). As a result of the moderated OLS regression, the interaction term between positive performance feedback and human capital is negatively associated with CEOs’ obsessive passion (Model 4, Table 2; *β* = -0.56, *p* < .05), failing to support Hypothesis 3. Fig 2 illustrates the moderated OLS regression results of the moderating effect of human capital on the relationship between positive performance feedback and obsessive passion.

**Fig 3:**
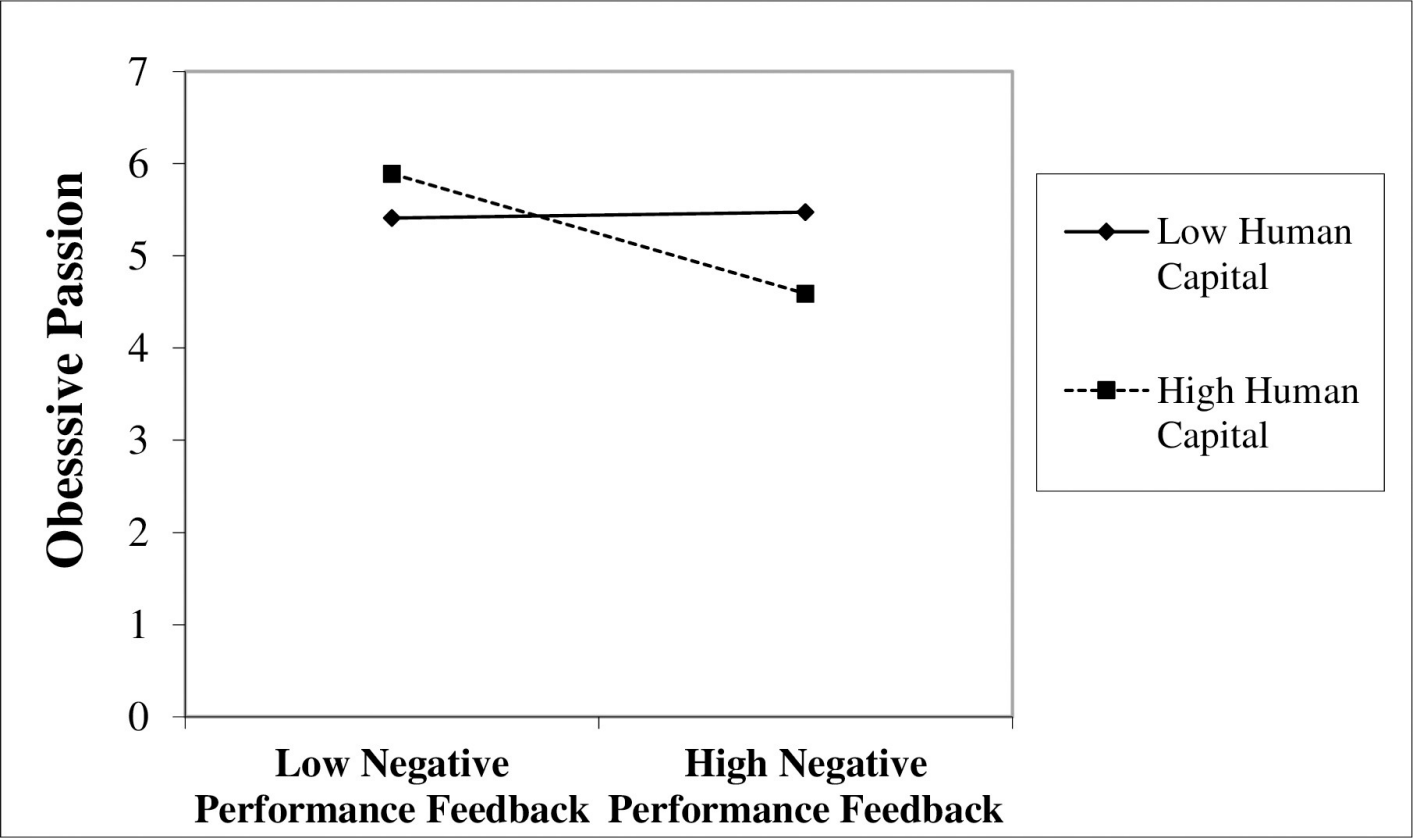
The moderating effect of human capital on the relationship between negative performance feedback and obsessive passion.

To test Hypothesis 4, we applied a sample of 83 CEOs with negative performance feedback. Table 3 displays the moderated OLS regression results for Hypothesis 4. Model 7 adds the moderating variable (i.e., human capital), and Model 8 contains the interaction variable (i.e., negative performance feedback × human capital). As a result of the moderated OLS regression, the interaction term between negative performance feedback and human capital is positively associated with CEOs’ obsessive passion (Model 8, Table 3; *β* = -0.74, *p* < .05), supporting Hypothesis 4. Fig 3 shows the moderated OLS regression results of the moderating effect of human capital on the relationship between negative performance feedback and obsessive passion.

### Post-hoc

We performed three additional tests as post-hoc to supplement the robustness of our results. First, acknowledging the existence of various types of passion, we tested for the influence of performance feedback on harmonious passion. In the second phase of the data collection, we collected data on harmonious passion using a six-item scale by Vallerand et al. [14]. Applying OLS regression, we found that both relationships (positive performance feedback and harmonious passion: *β* = 0.25, *p* = .08; negative performance feedback and harmonious passion: *β* = -0.22, *p* = .11) are insignificant. Although theoretically distinct types of passion, the results of post-hoc imply that performance feedback significantly impacts obsessive passion, not harmonious passion.

Second, we tested for the quadratic effects of performance feedback on obsessive passion. Empirical studies in the behavioral theory of the firm found curvilinear relationships between performance feedback and R&D intensity [9,88], firm growth [89], R&D investments [90], and organizational change [23]. In this regard, we tested for the potential existence of curvilinear relationships between performance feedback and obsessive passion; specifically, we tested if positive performance feedback would have a positive and increasing impact on obsessive passion, and if negative performance feedback would also have a positive and increasing influence on obsessive passion. To examine these quadratic relationships, we inserted squared terms of positive and negative performance feedback in the relevant regression models [91]. As a result, we found that both U-shaped relationships (positive performance feedback and obsessive passion: *β* = 0.23, *p* = .37; negative performance feedback and obsessive passion: *β* = 0.10, *p* = .68) are insignificant. In other words, the quadratic terms showed insignificant influences and did not alter the main results, implying that the curvilinear relationships do not exist.

Third, to supplement the different sample sizes for CEOs with positive (*N* = 106) and negative performance feedback (*N* = 83), we utilized bootstrapping-based methods through the PROCESS macro [92]. As a result, with 5,000 bootstrap samples, we found that both direct (positive performance feedback and obsessive passion: *β* = 0.55, *p* = .01; negative performance feedback and obsessive passion *β* = -0.55, *p* = .04), and moderation models (human capital → [positive performance feedback and obsessive passion]: *β* = -0.55, *p* = .03; human capital → [negative performance feedback and obsessive passion]: *β* = -0.53, *p* = .04) are statistically identical with the original results using the OLS regression.

## Discussion

In this paper, we examined how performance feedback influences CEOs’ passion. Specifically, we theorized and found that positive affective outcomes (performance above aspiration-level) increase obsessive passion, and negative affective outcomes (performance under aspiration-level) also advance obsessive passion. Positive performance feedback motivates CEOs to persist in their current domains and feel satisfied. As they experience positive outcomes, CEOs would increase obsessive passion toward their current domains. When CEOs face performance under aspiration-level, they expand problemistic search to fix current issues [8]. As such, our results imply that negative performance feedback advances CEOs’ obsessive passion due to their motivation to change the status quo. We theorized that negative emotions such as guilt aroused from performance discrepancy lead CEOs to take reparative actions [51,52], thereby nurturing obsessive passion. As the gap between the actual firm performance and CEOs’ aspirations widens, CEOs do not simply view lower-than-expected performance as a straightforward outcome. Instead, they intensify their drive for success and competitive aggressiveness, which can lead to the development of obsessive passion.

The influence of performance feedback on CEOs’ obsessive passion underscores a nuanced relationship between external achievements and internal motivations. Positive performance feedback, aligning with the behavioral theory of the firm, serves as an antecedent, increasing CEOs’ commitment and emotional engagement in their roles. This phenomenon aligns with the dualistic model of passion, where obsessive passion is driven by external rewards and recognition, leading to a heightened commitment towards achieving organizational goals [15]. However, this externally-driven passion may inadvertently neglect intrinsic satisfaction and long-term strategic planning, leading to an overemphasis on immediate accomplishments.

The impact of negative performance feedback on fostering obsessive passion suggests a complex adaptive response by CEOs. Contrary to the potential demotivating effects of negative feedback, our findings indicate that CEOs may redirect their dissatisfaction into a vigorous effort to bridge the gap between current performance and aspirational goals. This behavior can be interpreted as a form of resilience or a compensatory strategy, where the determination to surmount challenges and setbacks ignites passion. However, this type of passion, although advantageous in short-term crisis management and problem-solving, may result in stress and burnout over time, emphasizing the need for a balanced approach in feedback and goal-setting processes.

We hypothesized that human capital would positively moderate the relationship between positive performance feedback and CEOs’ obsessive passion. However, the empirical results show that human capital negatively moderates the influence of positive performance feedback on CEOs’ obsessive passion. Interestingly, the relationship between positive performance feedback and obsessive passion is intensified when firms have low levels of human capital. Firms with high human capital possess managers with various experience, managerial knowledge, coordination, and communication skills [58]. When CEOs satisfy with their performance outcomes and increase their obsessive passion, low human capital would vitalize the influence. This is because firms with low human capital are less likely to make exploratory changes and are also unlikely to bring new insights that could make them more flexible. Since low human capital diminishes organizational flexibility [58], firms with low human capital tend to be less receptive to new domains and are more inclined to make conservative decisions in familiar areas. This, in turn, further amplifies the impact of positive performance feedback on CEOs’ obsessive passion. As we hypothesized, the empirical results confirm that human capital positively moderate the relationship between negative performance feedback and obsessive passion.

This paper makes several critical contributions. First, this paper contributes to the behavioral theory of the firm by examining positive and negative performance feedback distinctively. We arbitrarily divided performance above and below aspiration-level to observe how positive and negative performance feedback impact CEOs’ obsessive passion. This arbitrary split enabled us to theoretically and empirically examine different antecedent roles of performance feedback types on passion. As Greve and Gaba [93] called for studies on the relationship between performance feedback and individual-level variables; this paper linked firm-level performance feedback and individual-level motivational factor. Specifically, we examined how different types of performance feedback (either positive or negative) would change CEOs’ level of passion. Moreover, we applied firm-level resource (i.e., human capital) as a moderator between performance feedback and obsessive passion. In this regard, we attempted to explain and find how firm-level factors influence individual-level affection. To further advance the behavioral theory of the firm, future studies should examine how problemistic search, slack, and exploratory behaviors are associated with passion. Based on the behavioral theory of firm, firms increase problemistic search when performance satisfaction decreases [8]. Also, firms with performance above aspiration-level develop exploratory behaviors such as R&D intensity [24]. In the extend, scholars could examine how performance feedback, problemistic search, exploratory behaviors, and passion are related.

Second, we advance the entrepreneurial passion literature by examining the antecedents of passion. Although outcomes of obsessive passion have been empirically studied, there is a lack of study in the antecedents [6,7]. Specifically, only limited studies have examined the formation of obsessive passion [33,34,94,95]. Understanding the antecedents of passion is vital for both individual- and organizational- success. At the individual-level, passion endows CEOs with the capacity for better decision-making in both their career and personal life choices, concurrently exerting a significant influence on their overall well-being [96]. While at the organizational-level, passion fuels innovation and cultivates resilience, which contributes to the firm’s growth [97,98]. Moreover, antecedent research serves as a cornerstone for effective leadership, facilitating team motivation, and elevating levels of employee engagement. As CEOs’ passion migrates to employees and gradually coalesces into an organizational culture [44], comprehending the determinants of passion becomes imperative for the success of the firm at a broader organizational-level. In this study, in response to the research call made regarding the relation between performance feedback and passion [39], we employed two distinct types of performance feedback as the antecedents of obsessive passion. Recognizing that the current state of research on antecedents remains in its nascent phase [6,7], a more nuanced understanding of these factors contributing to passion can provide invaluable insights to advance the literature.

Although not hypothesized, we found that CEOs’ obsessive passion would maintain when CEOs’ performance satisfaction- and aspiration-levels were similar. In other words, CEOs’ passion do not nurture when CEOs’ expectations on firm performance are met. This implies that variations in CEOs’ emotional responses to performance feedback play a pivotal role in the development of passion. As CEOs are driven by both positive and negative performance feedback, using it as motivation to invest more time and persist in efforts to change their current situation, further research is necessary to understand when passion diminishes, intensifies, and, most crucially, remains consistent, contingent upon their firm’s performance.

The current study has several limitations. First, part of the survey data on independent (i.e., performance satisfaction) and dependent variables (i.e., obsessive passion) are collected in the same period. Although we adopted a multiphase dataset and secured a 6-month gap to collect survey data on performance aspiration and satisfaction at two different time periods, there are limitations with causal inferences. As such, scholars should conduct a more delicate longitudinal research design to measure and examine similar models.

Second, this paper used survey data to measure performance feedback. We collected survey data on performance aspiration and satisfaction in two different time periods, and operationalized performance feedback by calculating the difference between performance satisfaction and aspiration-level. Although this method could enable us to observe whether satisfaction-level is above or below aspiration-level, all the measures are subjective. As such, future studies could apply same formula with objective dataset (e.g., financial documents) to measure performance feedback.

Third, although our research has identified human capital as a key boundary condition that reveals distinct patterns in the relation between performance feedback and passion, this relationship is further nuanced by various moderating and mediating factors, warranting additional studies to explore their influence. These potential moderating factors include industry characteristics (e.g., dynamism and munificence), internal organizational dynamics (e.g., culture and top management team compositions), organizational resources (slack and social capital), and CEO-specific factors (e.g., leadership, ownership, duality, and experience). Moreover, potential mediating factors include organizational decision-making processes and learning and adaptation. First, the complexity of decision-making in organizations, particularly within dominant coalitions [99], is a key mediator between performance feedback and passion. How this feedback is interpreted and integrated into decision-making processes by dominant coalitions can significantly influence organizational goals and strategies. This integration process, therefore, reshape the passion of CEOs, either enhancing or diminishing it based on the nature of the feedback and subsequent decisions. Second, organizational learning and adaptation in response to feedback, which can be either incremental or transformative, are crucial antecedents of CEO passion. This mediation occurs through how an organization processes feedback, leading to learning and changes that either enhance or rekindle passion among CEOs, influenced by the performance gap and capacity for change. These elements affect how feedback is interpreted and influence CEOs’ responses, underscoring the need for further studies on boundary conditions and mechanisms.

## Supporting information

S1 Appendix (DOCX)

S1 Data (SAV)
