# Supplementary material for: Performance feedback and obsessive passion: The moderating role of human capital
Source: PLoS One. 2024 Apr 18;19(4):e0302180. doi: 10.1371/journal.pone.0302180 (PMC11025744; doi:10.1371/journal.pone.0302180)
Supplement: S1 Appendix — (DOCX) [file pone.0302180.s001.docx]

**Appendix**

**List of the Survey**

**Independent variable**

- **Performance Feedback** (18 items) – Gupta & Govindarajan (1986)

**Moderating variable**

- **Human Capital** (9 items) – Jin et al. (2010)

**Dependent variable**

- **Obsessive Passion** (6 items) – Vallerand et al. (2003)

**Control variables**

- **Firm Age** (1 item)
- **Firm Size** (1 item)
- **Firm Type** (1 item)
- **Slack Resources** (4 items) – De Luca & Atuahene-Gima (2007)
- **CEO Gender** (1 item)
- **CEO Tenure** (1 item)
- **CEO Experience** (1 item)
- **CEO Effort** (1 item)
- **CEO Entrepreneurial Self-Efficacy** (4 items) – Zhao et al. (2005)

**List of the Full Survey Items**

**Independent variable**

- **Performance Feedback** (18 items) – Gupta & Govindarajan (1986). Gupta, A. K., & Govindarajan, V. (1986). Resource sharing among SBUs: Strategic antecedents and administrative implications. *Academy of Management Journal*, *29*(4), 695-714.

**Performance Aspiration** – Please indicate your level of aspiration for achieving each of the following performance criteria (1 = Minimal Aspirational Importance; 7 = Extremely High Aspirational Importance):

**Performance Satisfaction** – Please indicate your level of satisfaction regarding each of the following performance criteria (1 = Not at All Satisfied; 7 = Highly Satisfied):

1. Total Revenue
2. Sales Growth
3. Return on Assets
4. Return on Equity
5. Return on Investment
6. Operating Profits
7. Cash Flow
8. Market Share
9. Ability to Fund Growth from Profits

**Moderating variable**

- **Human Capital** (9 items) – Jin et al. (2010). Jin, Y., Hopkins, M. M., & Wittmer, J. L. (2010). Linking human capital to competitive advantages: Flexibility in a manufacturing firm’s supply chain. *Human Resource Management*, *49*(5), 939-963.

Please indicate the level of agreement with the following items (1 = Strongly Disagree; 7 = Strongly Agree):

1. Our managers have technical knowledge that is relevant to their responsibilities.
2. Our managers have general people management skills (planning, organizing, directing, evaluating, and motivating).
3. Our managers have knowledge of the strengths and weaknesses of our firm.
4. Our managers have the necessary coordination skills to work well with other departments in our firm.
5. Our managers have the necessary coordination skills to work well with our suppliers.
6. Our workers have multiple technological skills.
7. Our workers have problem-solving skills.
8. Our workers have the necessary interpersonal skills to work well with their coworkers.
9. Our workers have experience that is relevant to their jobs.

**Dependent variable**

- **Obsessive Passion** (6 items) – Vallerand et al. (2003). Vallerand, R. J., Blanchard, C., Mageau, G. A., Koestner, R., Ratelle, C., Léonard, M., ... & Marsolais, J. (2003). Les passions de l’ame: On obsessive and harmonious passion. *Journal of Personality and Social Psychology*, *85*(4), 756-767.

Please indicate the level of agreement with the following items (1 = Strongly Disagree; 7 = Strongly Agree):

1. I have difficulties controlling my urge to do my work.
2. I have almost an obsessive feeling for my work.
3. My work is the only thing that really turns me on.
4. If I could, I would only do my work.
5. My work is so exciting that I sometimes lose control over it.
6. I have the impression that my work controls me.

**Control variables**

- **Firm Age** (1 item) – Firm establishment year
- **Firm Size** (1 item) – Number of employees
- **Firm Type** (1 item) – 1) Public 2) Private 3) Other
- **Slack Resources** (4 items) – De Luca & Atuahene-Gima (2007). De Luca, L. M., & Atuahene-Gima, K. (2007). Market knowledge dimensions and cross-functional collaboration: Examining the different routes to product innovation performance. *Journal of Marketing*, *71*(1), 95-112.

Please indicate the level of agreement with the following items (1 = Strongly Disagree; 7 = Strongly Agree):

1. Our firm has uncommitted resources that can be used to fund strategic initiatives at short notice.
2. Our firm has a large amount of resources available in the short run to fund our initiatives.
3. Our firm will have no problems obtaining resources at short notice to support new strategic initiatives.
4. Our firm has a large amount of resources at the discretion of management to fund new strategic initiatives.

- **CEO Gender** (1 item) – 0) Female 1) Male
- **CEO Tenure** (1 item) – Have been in the company for ______ years.
- **CEO Experience** (1 item) – A previous experience in starting a new business 0) No 1) Yes
- **CEO Effort** (1 item) – Please indicate the level of effort you have invested in your work over the past year (1 = Significantly Below Goals; 7 = Significantly Above Goals).
- **CEO Entrepreneurial Self-Efficacy** (4 items) – Zhao et al. (2005). Zhao, H., Seibert, S. E., & Hills, G. E. (2005). The mediating role of self-efficacy in the development of entrepreneurial intentions. *Journal of Applied Psychology*, *90*(6), 1265-1272.

Please indicate the level of confidence with the following items (1 = Not Very Confident; 7 = Very Confident):

1. Identifying new business opportunities.
2. Creating new products or services.
3. Thinking creatively.
4. Commercializing an idea or new development.
